# Pharmacological Treatment of Mood Disorders and Comorbid Addictions: A Systematic Review and Meta-Analysis: Traitement Pharmacologique des Troubles de L’humeur et des Dépendances Comorbides: Une Revue Systématique et une Méta-Analyse

**DOI:** 10.1177/0706743720915420

**Published:** 2020-04-17

**Authors:** Paul R. A. Stokes, Tahir Jokinen, Sami Amawi, Mutahira Qureshi, Muhammad Ishrat Husain, Lakshmi N. Yatham, John Strang, Allan H. Young

**Affiliations:** 1 Department of Psychological Medicine, Centre for Affective Disorders, 34426 Institute of Psychiatry, Psychology and Neuroscience, King’s College London, United Kingdom; 2 South London and Maudsley NHS Foundation Trust, Beckenham, Kent, United Kingdom; 3 National Institute for Health Research (NIHR) Maudsley Biomedical Research Centre (BRC) at South London Maudsley Foundation Trust and King’s College London, United Kingdom; 4 Department of Psychiatry, University of Toronto, Canada; 5 Centre for Addiction and Mental Health, Toronto, Canada; 6 8166 University of British Columbia, Vancouver, Canada; 7 Department of Addictions, 34426 Institute of Psychiatry, Psychology and Neuroscience, King’s College London, United Kingdom

**Keywords:** bipolar disorders, major depressive disorder, addictions, pharmacotherapy, antidepressants, comorbidity, meta-analysis, randomized controlled trial, systematic reviews

## Abstract

**Objective:**

Addiction comorbidity is an important clinical challenge in mood disorders, but the best way of pharmacologically treating people with mood disorders and addictions remains unclear. The aim of this study was to assess the efficacy of pharmacological treatments for mood and addiction symptoms in people with mood disorders and addiction comorbidity.

**Methods:**

A systematic search of placebo-controlled randomized controlled trials investigating the effects of pharmacological treatments in people with bipolar disorder (BD) or major depressive disorder (MDD), and comorbid addictions was performed. Treatment-related effects on mood and addiction measures were assessed in a meta-analysis, which also estimated risks of participant dropout and adverse effects.

**Results:**

A total of 32 studies met systematic review inclusion criteria. Pharmacological therapy was more effective than placebo for improving manic symptoms (standardized mean difference [SMD] = −0.15; 95% confidence interval [95% CI], −0.29 to −0.02; *P* = 0.03) but not BD depressive symptoms (SMD = −0.09; 95% CI, −0.22 to 0.03; *P* = 0.15). Quetiapine significantly improved manic symptoms (SMD = −0.23; 95% CI, −0.39 to −0.06; *P* = 0.008) but not BD depressive symptoms (SMD = −0.07; 95% CI, −0.23 to 0.10; *P* = 0.42). Pharmacological therapy was more effective than placebo for improving depressive symptoms in MDD (SMD = −0.16; 95% CI, −0.30 to −0.03; *P* = 0.02). Imipramine improved MDD depressive symptoms (SMD = −0.58; 95% CI, −1.03 to −0.13; *P* = 0.01) but Selective serotonin reuptake Inhibitors (SSRI)-based treatments had no effect (SMD = −0.06; 95% CI, −0.30 to 0.17; *P* = 0.60). Pharmacological treatment improved the odds of alcohol abstinence in MDD but had no effects on opiate abstinence.

**Conclusions:**

Pharmacological treatments were significantly better than placebo in improving manic symptoms, MDD depressive symptoms, and alcohol abstinence but were not better for bipolar depression symptoms. Importantly, quetiapine was not more effective than placebo in improving bipolar depression symptoms nor were SSRI’s for the treatment of MDD depression. Our findings highlight the need for further high-quality clinical trials of treatments for mood disorders and comorbid addictions.

## Abstract (fr)

**Objectif:**

La comorbidité de la dépendance est un important problème clinique dans les troubles de l’humeur, mais la meilleure façon de traiter les personnes souffrant de troubles de l’humeur et de dépendances en pharmacologie demeure indéfinie. Cette étude visait à évaluer l’efficacité des traitements pharmacologiques pour les symptômes de l’humeur et de dépendance au sein des troubles de l’humeur avec comorbidité de la dépendance.

**Méthodes:**

Une recherche systématique des essais randomisés contrôlés par placebo et recherchant les effets des traitements pharmacologiques chez les personnes souffrant de trouble bipolaire (TB) ou de trouble dépressif majeur (TDM) et de dépendances comorbides a été menée. Les effets liés au traitement sur les mesures de l’humeur et de la dépendance ont été évalués dans une méta-analyse qui estimait également les risques d’abandon des participants et les effets indésirables.

**Résultats:**

Trente-deux études satisfaisaient aux critères d’inclusion de la revue systématique. La thérapie pharmacologique était plus efficace que le placebo pour améliorer les symptômes de manie (différence moyenne normalisée DMN = −0.15; IC à 95% −0.29 à −0.02; *P* = 0.03) mais pas les symptômes dépressifs du TB (DMN = −0.09; IC à 95% −0.22 à 0,03; *P* = 0.15). La quétiapine améliorait significativement les symptômes de manie (DMN = −0.23; IC à 95% −0.39 à −0.06; *P* = 0.008) mais pas les symptômes dépressifs du TB (DMN = −0.07; IC à 95% −0.23 à 0.10; *P* = 0.42). La thérapie pharmacologique était plus efficace que le placebo pour améliorer les symptômes dépressifs du TDM (DMN = −0.16; IC à 95% −0.30 à −0.03; *P* = 0.02). L’imipramine améliorait les symptômes dépressifs du TDM (DMN = −0.58; IC à 95% −1.03 à −0.13; *P* = 0.01) mais les traitements à base d’ISRS n’avaient aucun effet (DMN = −0.06; IC à 95% −0.30 à 0.17; *P* = 0.60). Le traitement pharmacologique améliorait les probabilités d’abstinence d’alcool dans le TDM mais n’avait aucun effet sur l’abstinence d’opiacés.

**Conclusions:**

Les traitements pharmacologiques étaient significativement meilleurs que les placebos pour améliorer les symptômes de manie, les symptômes dépressifs du TDM et l’abstinence d’alcool, mais n’étaient pas plus efficaces pour les symptômes de la dépression bipolaire. Notablement, la quétiapine n’était pas plus efficace que le placebo pour améliorer les symptômes de la dépression bipolaire, pas plus que ne l’étaient les ISRS pour le traitement de la dépression du TDM. Nos résultats mettent en lumière le besoin d’autres essais cliniques de grande qualité sur les traitements des troubles de l’humeur et des dépendances comorbides.

## Introduction

Addiction comorbidity is a major challenge for the treatment of mood disorders, particularly for bipolar disorder (BD).^1^ People with mood disorders, such as BD or major depressive disorder (MDD), are at an increased risk of developing addiction comorbidity. The recent U.S. National Epidemiologic Survey on Alcohol and Related Conditions found that people with bipolar I disorder experience a 2.3 times increased lifetime prevalence rate of substance use disorder after adjusting for other psychiatric comorbidities,^2^ and people with MDD experience a 1.5 times increased rate of substance use disorders.^3^ People with mood disorders are also at increased risks of behavioral addictions, such as gambling disorder, and 1 in 10 people with BD may have a moderate to severe lifetime risk of problem gambling.^4^ Addiction comorbidity in mood disorders is important as it detrimentally impacts on illness burden and clinical outcomes. In both BD and MDD, addiction comorbidity is associated with increased risks of suicide, lower remission rates, increased severity of mood symptoms,^5–7^ and in BD increased risks of violence during a manic episode.^8^

Although rates of addiction comorbidity are clearly elevated in mood disorders, the behavioral and neurobiological mechanisms that mediate increased risk are not well understood. One possibility is that people with mood disorders self-medicate with alcohol and other substances to reduce the burden of mood symptoms.^9,10^ Another possibility is that there is a shared neurobiology underpinning both mood disorders and addictions potentially mediated by early life stress and/or deficits in neurotransmitter function.^11–13^ As so little is known about the reasons for the increased prevalence of addiction comorbidity in mood disorders, it is difficult to rationally implement targeted pharmacological treatments. Despite the importance of addiction comorbidities for the treatment of mood disorders, the most effective way of pharmacologically treating people with mood disorders and addictions remains unclear. The aim of this systematic review and meta-analysis is to provide a comprehensive review of the efficacy of pharmacological treatments for mood disorders and addiction comorbidities and to determine the effect size of treatments on mood and addiction symptoms.

## Methods

### Study Identification and Selection

A systematic review protocol was developed by 2 of the authors (P.R.S. and T.J.). Five bibliographical and clinical trial databases Medline, PubMed, PsychINFO, EMBASE, and the Cochrane Central Register of Controlled Trials were searched from inception until September 19, 2017. Studies were required to be available in English. Each search was conducted by 2 researchers (T.J. and S.A.), independent of each other, and discrepancies were discussed and agreed with a third researcher (P.R.S.). The database searches were used to identify double-blind placebo-controlled randomized controlled trials (RCTs) published in peer-reviewed journals, which investigated the effects of pharmacological treatments in participants with mood disorders with addiction comorbidity, aged 18 years and older. Citations of relevant studies and reviews were checked to identify papers missed in the initial database search.

The following search string was used: (depressi*[Title/Abstract] OR *bipolar*[Title/Abstract] OR *mania*[Title/Abstract] OR *manic*[Title/Abstract]) AND (addicti*[Title/Abstract] OR *dependence*[Title/Abstract] OR *“substance use”*[Title/Abstract] OR *“substance misuse”*[Title/Abstract] OR *“substance abuse”*[Title/Abstract] OR alcoho*[Title/Abstract] OR *“substance related”*[Title/Abstract] OR *“substance-related”*[Title/Abstract] OR *“drug abuse”*[Title/Abstract] OR *“drug disorder”*[Title/Abstract] OR *“drug misuse”*[Title/Abstract] OR *nicotine*[Title/Abstract] OR smok*[Title/Abstract] OR *cigarette$*[Title/Abstract] OR *tobacco*[Title/Abstract] OR *gambling*[Title/Abstract] OR *cocaine*[Title/Abstract] OR *cannabis*[Title/Abstract] OR *marijuana*[Title/Abstract] OR *“recreational drug$”*[Title/Abstract]) AND (*randomi! ed controlled trial* OR *randomi! ed control trial* OR *random controlled trial* OR *random control trial* OR *RCT* OR *“clinical trial”*).

### Inclusion and Exclusion Criteria

Studies were included if participants had a diagnosis of bipolar I, bipolar II, or MDD and substance abuse, substance dependence, substance use disorder, or pathological gambling according to *International Classification of Diseases* or *Diagnostic and Statistical Manual of Mental Disorders* criteria. Pathological gambling, now termed gambling disorder, was included as it is recognized as a substance-related and addictive disorder within *Diagnostic and Statistical Manual of Mental Disorders, Fifth Edition*,^14^ has behavioral similarities to substance addiction,^15^ and people with BD are at a substantially increased risk of developing gambling problems.^4^ Non-placebo-controlled studies, open-label trials, case reports, reviews, and studies where the mood disorder diagnosis was secondary to substance use were excluded. Concomitant medications and psychological therapies were permitted as long as the primary treatment was pharmacological. Only studies that reported mean end-of-study mood or addiction outcome variables or mean percentage change in these variables from baseline to end of study were included in the meta-analysis.

### Outcome Measures

The primary outcome of included studies was the effect of drug treatment on mood and addiction symptoms. For mood symptoms, treatment effects were measured by end-of-trial scores, or percentage change in these scores from baseline to end point, on validated mood rating scales. Treatment effects on addiction symptoms were measured by daily reported end-of-trial substance consumption and by end-of-trial rates of abstinence, or percentage change in these measures from baseline to end point. Secondary outcome measures included the frequency of participant dropouts, serious adverse events (SAEs), and frequency of psychiatric adverse events (AEs). The Clinicaltrials.gov online database was additionally checked for studies where mean end-of-study mood or addiction outcome variables or mean percentage change in these variables was not reported in the study manuscript.

### Quality Assessment

Quality of studies included in the systematic review was assessed using the Effective Public Health Practice Project Quality Assessment Tool^16^ by 2 researchers (M.I.H. and M.Q.) independent from those who identified and selected studies. Study data quality was assessed in 6 domains: presence of selection bias, strength of study design, presence of confounders, blinding of outcome assessors, strength of data collection methods, and reporting of withdrawals and dropouts. The domain scores were then combined to generate a global quality rating for each study. Discrepancies in global rating scores were discussed between the 2 researchers and a consensus score agreed.

### Statistical Analysis

The meta-analysis and all statistical analyses were conducted using Review Manager (RevMan) Version 5.3.^17^ Standardized mean difference (SMD) effect sizes, also known as Hedges (adjusted) g, were calculated for continuous outcome measures, such as mood scores, using a random effects model.^18^ Heterogeneity of analyses was assessed using the *I* ^2^ statistic, which indicates the proportion of effect size variance likely due to study heterogeneity.^19^ The odds ratio (*OR*) of abstinence and the relative risk (RR) of participant dropout and AEs were calculated using a Mantel–Haenszel statistical method with a random effects analysis model. Publication bias was assessed by visual inspection of a Funnel plot^20^ and by calculating Eggers regression test for publication bias^21^ using meta-analytical equations entered into Excel (see www.ptsdmri.uk).^22^

## Results

**Figure 1.**
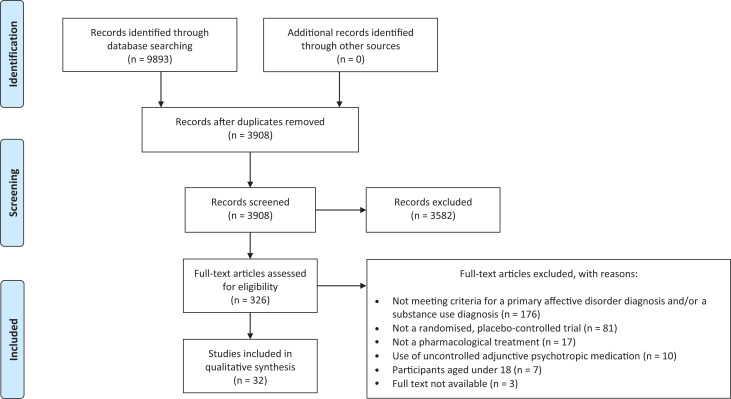
Preferred reporting items for systematic reviews and meta-analyses flow diagram of search results.^23^

The initial search identified 9,886 papers. After exclusion of duplicates and initial screening by title and abstract, 319 articles remained. Following assessment, 32 papers were included in the final analysis, 13 studies examining treatment effects in participants with BD (1,093 participants) and 19 in MDD (1,849 participants); see Figure 1 for details of papers excluded. The Dorus et al. study did not explicitly clarify whether the diagnosis of MDD was secondary to alcohol use,^24^ but this study was included as participants with a history of depression and alcohol dependency scored in the mild to moderate depression severity range at baseline after 3 weeks of abstinence from alcohol.

The included study durations ranged from 29 days to 52 weeks (mean 13.5 weeks ± 7.9) and participant sample size ranged from 5 to 345 (treatment group mean participant sample size 47.4 ± 45.6), although most studies were small, with only 12 having sample sizes greater than 50. All studies used nonactive placebo comparators. For the BD studies, 7 studies investigated treatment effects in alcohol addiction comorbidity,^25–31^ 5 in stimulant addiction comorbidity,^32–36^ and 1 in gambling disorder comorbidity (see Table 1).^37^ For the MDD studies, 13 studies investigated treatment effects in alcohol addiction comorbidity^24,38–49^ and 6 investigated treatment effects in other addiction comorbidities including cocaine, opiates, and nicotine addictions^50–55^ (see Table 2). Two of the BD studies^25,33^ and 5 of the MDD studies^24,38,40,46,51^ examined treatment effects in participants who were abstinent from substance use at the beginning of studies. One BD^28^ and 6 MDD studies^50–52,55^ were excluded from the meta-analysis as they did not report the required outcome measures.

**Table 1.** Placebo-Controlled Randomized Clinical Trials Examining Response to Pharmacological Treatment in Participants with Bipolar Disorder and Comorbid Addictions.

Study	Mood Disorder	Addiction Disorder and Abstinence Status at Start of Study	Treatment	Study Duration	Treatment/Placebo group sizes	Mood Scales Used	Completion Rates	Psychotherapy Used?	Results
Sylvia et al. (2016)^30^	Bipolar I or II (*DSM-IV*)	Alcohol dependence (*DSM-IV*), not abstinent	Topiramate 150 mg BD adjunctive to current medication	12 Weeks	5/7	HAM-D and YMRS	40% Topiramate, 71% placebo	Yes	Topiramate associated with improvement in depression and manic symptoms but no improvement in drinking behavior.
Brown et al. (2015)^56^	Bipolar I (*DSM-IV*)	Cocaine dependence (*DSM-IV*), not abstinent	Citicoline 2,000 mg/day adjunctive to current medication	12 Weeks	61/61	HAM-D and YMRS	Citicoline 71%, placebo 57%	Yes	Citicoline initially effective for cocaine use but effect diminishes over time and no significant effect on mood scores.
Brown et al. (2014)^29^	Bipolar I or II (*DSM-IV*)	Alcohol dependence (*DSM-IV*), not abstinent	Quetiapine 600 mg/day adjunctive to a mood stabilizer	12 Weeks	44/44	HAM-D and YMRS	98% in both groups combined	Yes	No significant effect on mood measures or alcohol consumption.
Brown and Gabrielson (2012)^35^	Bipolar I, II, or MDD (*DSM-IV*)	Amphetamine dependence (*DSM-IV*) with methamphetamine use, not abstinent	Citicoline 2,000 mg/day adjunctive to current medication	12 Weeks	28/20	IDS-C. Mania not assessed	Citicoline 41%, placebo 14%	Not stated	Citicoline associated with improvement in depression symptoms but no significant effects on methamphetamine use.
Brown et al. (2012)^36^	Bipolar I or II or BD-NOS or cyclothymic disorder (*DSM-IV*)	Cocaine dependence (self-reported), not abstinent	Lamotrigine 400 mg/day adjunctive to current medication	10 Weeks	55/57	HAM-D and YMRS	52% completed overall, completion rates similar between groups	Not stated	Lamotrigine associated with a reduction in spending on cocaine but no significant effect on positive urine screens or mood symptoms
Tolliver et al. (2012)^31^	Bipolar I or II (*DSM-IV*)	Alcohol dependence (*DSM-IV*), not abstinent	Acamprosate 2,000 mg/day augmentation of citalopram	14 Weeks	14/16	MADRS and YMRS	Acamprosate 75%, placebo 65%	Yes	No significant effects on drinking outcomes or mood scores.
Brown et al., 2010^34^	Bipolar I or II (*DSM-IV*)	Cocaine dependence (*DSM-IV*), not abstinent	Quetiapine 400 to 800 mg/day	12 Weeks	7/5	HAM-D and YMRS	Quetiapine 86%, placebo 40%	Not stated	No significant effects on cocaine use or craving or on mood measures
Stedman et al. (2010)^26^	Bipolar I (*DSM-IV*)	Alcohol dependence (*DSM-IV*), not abstinent	Quetiapine 300 to 800 mg/day adjunctive to lithium or divalproex	12 Weeks	159/169	MADRS and YMRS	Quetiapine 42%, placebo 43%	No	No significant effects on alcohol consumption or mood scores.
Brown et al. (2009)^28^	Bipolar I or II (*DSM-IV*)	Alcohol dependence (*DSM-IV*), not abstinent	Naltrexone 50 mg/day adjunctive to current medication	12 Weeks	20/23	HAM-D and YMRS	Naltrexone 70%, placebo 52%	Yes	Naltrexone associated at trend significance level with an improvement in alcohol consumption and craving but no effect on mood scores.
Brown et al. (2008)^27^	Bipolar I disorder and MDD (*DSM-IV*)	Alcohol abuse or dependence (*DSM-IV*), not abstinent	Quetiapine 600 mg/day adjunctive to current medication	12 Weeks	102/50	HAM-D and YMRS	Not stated	No	No significant effect on alcohol use or manic symptoms. Depression symptoms improved significantly in quetiapine group.
Brown et al. (2007)^33^	Bipolar I and II disorders (*DSM-IV*)	Cocaine dependence, abstinent 1 to 12 weeks before study entry	Citicoline up to 2 g/day adjunctive to current medication	12 Weeks	44/21	IDS-SR and YMRS	39% Citicoline and 19% placebo	Not stated	Citicoline associated with improvement in aspects of declarative memory and cocaine use but not mood scores.
Salloum et al. (2005)^25^	Bipolar I (*DSM-IV*)	Alcohol dependence (*DSM-IV*), participants randomized after 1-week substance detoxification	Valproate 750 mg/day adjunctive to lithium	24 Weeks	27/25	HAM-D and BRMS	44% valproate, 32% placebo	Yes	Valproate associated with significant improvements in proportion of heavy drinking days but not in mood symptoms.
Hollander et al. (2005)^37^	Bipolar II or cyclothymia (*DSM-IV*)	Pathological gambling (*DSM-IV*), abstinence status not stated	Lithium sustained release 300 mg TDS	10 Weeks	12/17	HAM-D and CARS-M	67% lithium, 77% placebo	No	Lithium associated with improvements in pathological gambling and mania symptoms and with a trend improvement in depression symptoms.

*Note*. BD = bipolar disorder; BD-NOS = bipolar disorder–not otherwise specified; BRMS = Bech-Rafaelsen Mania Scale; CARS-M = Clinician-Administered Rating Scale for Mania; *DSM-IV* = *Diagnostic and Statistical Manual of Mental Disorders, Fourth Edition*; HAM-D = Hamilton Depression Rating scale; IDS-C = Clinician-rated Inventory of Depressive Symptomatology; IDS-SR = Inventory of Depressive Symptomatology—Self Report; MDD = major depressive disorder; TDS = total dissolved solids; YMRS = Young Mania Rating Scale.

**Table 2.** Placebo-Controlled Randomized Clinical Trials Examining Response to Pharmacological Treatment in Participants with Major Depression and Comorbid Addictions.

Study	Mood Disorder	Addiction Disorder and Abstinence Status at Start of Study	Treatment	Study Duration	Treatment/Placebo Group Sizes	Mood Scale Used	Completion Rate	Psychotherapy Used?	Results
Cornelius et al. (2016)^42^	MDD (*DSM-IV*)	Alcohol use disorder (*DSM-IV*), not abstinent	Mirtazapine 30 mg/day	12 Weeks	7/7	BDI	Not stated	Yes	No improvements in alcohol consumption or depression symptoms.
Adamson et al. (2015)^48^	MDD (*DSM-IV*)	Alcohol dependence (*DSM-IV*), not abstinent	Citalopram 20 to 60 mg/day adjunctive to naltrexone 50 to 100 mg/day	12 Weeks	138/65	MADRS	75% of overall sample completed	Yes	No significant effect on depression symptoms or alcohol consumption measures.
Raby et al. (2014)^54^	MDD or dysthymia (*DSM-III-R*)	Cocaine dependence (*DSM-III-R*), abstinence status not reported	Venlafaxine 300 mg/day	12 Weeks	64/66	HAM-D	Venlafaxine 33%, placebo 49%	Yes	Venlafaxine not superior to placebo on either mood or cocaine use outcomes.
Levin et al. (2013)^55^	MDD or dysthymia (*DSM-IV*)	Cannabis dependence (*DSM-IV*), abstinence status not reported	Venlafaxine extended release max 375 mg/day	12 Weeks	51/52	HAM-D	62% of overall sample completed	Yes	No significant difference between groups in mood improvement. Cannabis abstinence rates worse in venlafaxine group.
Ralevski et al. (2013)^47^	MDD (*DSM-IV*)	Alcohol dependence (*DSM-IV*), not abstinent	Mecamylamine 10 mg/day	12 Weeks	11/10	HAM-D	Mecamylamine 36%, 80% placebo	Unknown	No significant effect on mood scores or alcohol abstinence. Mecamylamine improved alcohol consumption in nonsmokers.
Witte et al. (2012)^44^	MDD (*DSM-IV*)	Alcohol abuse or dependence (*DSM-IV*), not abstinent	Acamprosate 2,000 mg/day adjunctive to escitalopram 30 mg/day	12 Weeks	12/11	HAM-D	Acamprosate 58%, placebo 45%	Yes	No significant effect on depression symptoms or alcohol consumption.
Pettinati et al. (2010)^38^	Major depression (*DSM-IV*)	Alcohol dependence (*DSM-IV*), abstinent for 3 consecutive days	Sertraline 200 mg/day and naltrexone 100 mg/day, naltrexone 100 mg/day, sertraline 200 mg/day, all adjunctive to current medication	14 Weeks	42, 49, 40/39	HAM-D	Sertraline and naltrexone 57%, naltrexone 59%, sertraline 52%, placebo 59%	Yes	Significantly higher rates of alcohol abstinence in the sertraline and naltrexone groups. Number of participants in the sertraline and naltrexone groups not depressed at the end of treatment approached significance compared with the other treatment groups.
Petrakis et al. (2007)^40^	Major depression (*DSM-IV*)	Alcohol dependence (*DSM-IV*), abstinent for ≤29 days	Naltrexone 50 mg and disulfiram 250 mg, disulfiram 250 mg, naltrexone 50 mg	12 Weeks	28, 43, 34/34	HAM-D	Not stated	Not stated	No relationship between diagnosis of depression and treatment on alcohol consumption or depression symptoms.
Kranzler et al. (2006)^39^	MDD (*DSM-IV*)	Alcohol dependence (*DSM-IV*), not abstinent	Sertraline 200 mg	10 Weeks	160/171	HAM-D	Group A (HAM-D ≥ 17) 58.7%, placebo 56.0%. Group B (HAM-D ≤16) 55.7%, placebo 78.3%	Not stated	No significant effects on depression symptoms or drinking behavior.
Hernandez-Avila et al. (2004)^43^	Major depression (*DSM-IV*)	Alcohol dependence (*DSM-IV*), not abstinent	Nefazodone 300 mg/day	10 Weeks	21/20	HAM-D	Nefazodone 62%, placebo 75%	Yes	No significant improvements in depressive or anxiety symptoms. Nefazodone treatment associated with a reduction in alcohol consumption.
Gual et al. (2003)^46^	MDD or dysthymia (*DSM-IV*)	Alcohol dependence (*DSM-IV*), participants required to remain abstinent for ≥2 weeks following detoxification	Sertraline 50 to 150 mg/day	24 Weeks	44/39	HAM-D	Sertraline 55%, placebo 56%	Not stated	No significant effect on drinking outcome measures or depression scores.
Schmitz et al. (2001)^52^	MDD (*DSM-IV*)	Cocaine dependence (*DSM-IV*), not abstinent	Fluoxetine 40 mg/day	12 Weeks	34/34	HAM-D	Fluoxetine 53%, 41% placebo	Yes	Fluoxetine not associated with significant improvements in depressive symptoms or cocaine use.
Thorsteinsson et al. (2001)^51^	MDD (*DSM-III-R*)	Cigarette smoking (≥1 pack per day for at least 1 year), abstinent when starting transdermal patch	Transdermal nicotine 21 mg/24 hr	29 days	18/20	HAM-D	63% of overall sample completed	Yes	Significantly fewer participants resumed smoking in the nicotine group. No significant effect on mood scores.
Roy-Byrne et al. (2000)^41^	MDD (*DSM-III-R*)	Alcohol dependence (*DSM-III-R*), not abstinent	Nefazodone 500 mg/day	12 Weeks	32/32	HAM-D	Nefazodone 62%, 34% placebo	Yes	Nefazodone associated with significantly lower depression scores, collapsed over all time points, but no differences in alcohol consumption.
Nunes et al. (1998)^53^	MDD or dysthymia (*DSM-III-R*)	Patients receiving methadone maintenance treatment, not abstinent	Imipramine hydrochloride 300 mg/day in addition to methadone	12 Weeks	42/42	HAM-D	57% imipramine, 67% placebo	Yes	Depression symptoms and craving significantly lower after treatment with imipramine compared with placebo.
Cornelius et al. (1997)^49^	MDD (*DSM-III-R*)	Alcohol dependence (*DSM-III-R*), not abstinent	Fluoxetine 20 to 40 mg/day	12 Weeks	25/26	HAM-D	90% of overall sample completed	Yes	Fluoxetine treatment associated with reduction in depressive symptoms and alcohol consumption.
McGrath et al. (1996)^45^	MDD or dysthymia (*DSM-III-R*)	Alcohol dependence or abuse (*DSM-III-R*), not abstinent	Imipramine hydrochloride 300 mg/day	12 Weeks	36/33	HAM-D	81% of overall sample completed	Yes	Imipramine treatment associated with improvement in depression symptoms. Imipramine may improve alcohol consumption in participants whose depression responded to treatment.
Dorus et al. (1989)^24^	Major depression or dysthymic disorder (*DSM-III*)	Alcohol dependence (*DSM-III*), ≥ 3 weeks of abstinence	Lithium 1,200 mg/day	52 Weeks	89/82	BDI	Lithium 62%, placebo 65%	Yes	No significant effect of lithium on severity of depression, alcohol consumption or abstinence rates.
Kleber et al. (1983)^50^	Major depression (*DSM-II*)	Opiate dependence on methadone, abstinence status not reported	Imipramine up to 225 mg	8 Weeks	23/23	HAM-D	Imipramine 57%, 48% placebo	Yes	Imipramine treatment not associated with improvements in depression symptoms or drug use.

*Note*. BDI = Beck Depression Inventory; DSM-II = *Diagnostic and Statistical Manual of Mental Disorders*, *Second Edition; DSM-III-R* = *Diagnostic and Statistical Manual of Mental Disorders, Third Edition, Revised; DSM-IV = Diagnostic and Statistical Manual of Mental Disorders, Fourth Edition*; HAM-D = Hamilton Depression Rating scale; MADRS = Montgomery-Åsberg Depression Rating Scale; MDD = major depressive disorder.

Only one study, Hollander et al., investigated the effect of treatment in a mood disorders and gambling disorder comorbidity population. This study found, in people with a bipolar spectrum disorder and pathological gambling treated with lithium 900 mg/day, a significant improvement in mania scores (SMD = −0.77) and an improvement in depression scores at a trend significance level (SMD = −0.73).^37^ This study was not included in the meta-analysis to reduce heterogeneity of comorbid patient populations, and so the meta-analysis scope was restricted only to alcohol and other substance addiction comorbidities.

### Quality and Bias Assessment

Quality assessment results are summarized in Supplemental Table 1. Ten studies were assessed as having a strong quality rating,^25,28,31,38,40,43,48,53–55^ 15 were assessed as having a moderate quality rating,^24,26,27,29,30,33,35–37,44,46,49,50,52,56^ and 7 were assessed as having a weak quality rating.^34,39,41,42,45,47,51^

The Funnel plot (see Supplemental Figure 1) was asymmetrical suggesting that studies without statistically significant effects remain unpublished, particularly those with small participant sample sizes and those that investigated treatment effects in BD. Eggers regression test indicated significant publication bias (*P* = 0.02).

### Meta-Analysis: Effects on Mood Scores

#### Treatment effects on mania scores

**Figure 2.**
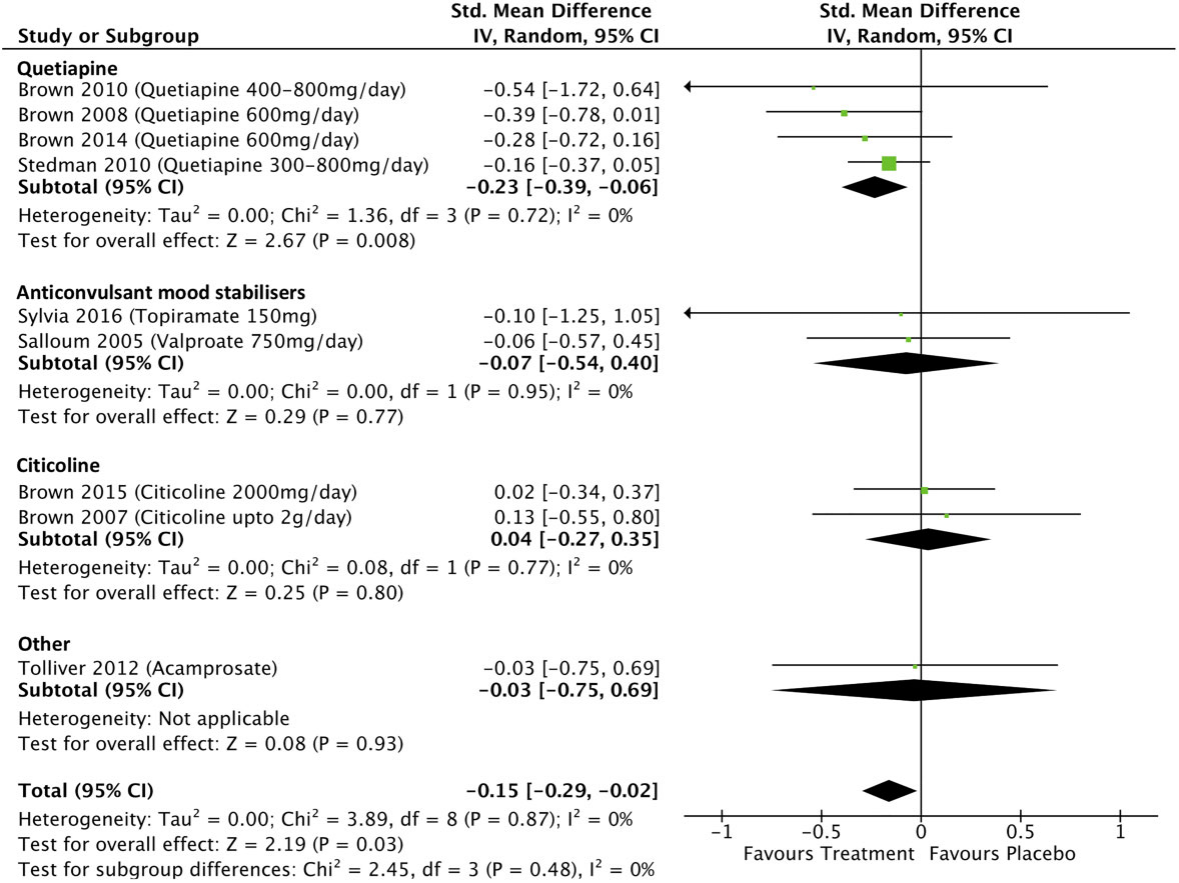
Treatment effects on mania scores in bipolar disorder with addiction comorbidity.

Data from 9 studies which investigated the effects of treatment on mania scores in people with BD with addiction comorbidities were included in the meta-analysis. The overall pooled effect size (SMD) for these were −0.15 (95% confidence interval [95% CI], −0.29 to −0.02; *P* = 0.03; *I* ^2^ = 0%; see Figure 2). The effects of quetiapine on mania scores were examined in 4 studies, and the meta-analysis found a significant effect of treatment with a pooled effect size of −0.23 (95% CI, −0.39 to −0.06; *P* = .008; *I* ^2^ = 0%). Omitting one quetiapine study which was assessed as weak quality^34^ from the analysis changed the pooled effect size to −0.22 (−0.39 to −0.05; *P* = 0.01; *I* ^2^ = 0%). The effects of anticonvulsant mood stabilizers on mania scores were examined in 2 studies with a pooled effect size of −0.07 (95% CI, −0.54 to 0.40; *P* = 0.77; *I* ^2^ = 0%), and 2 studies examined the effects of citicoline on mania scores with a pooled effect size of 0.04 (95% CI, −0.27 to 0.35; *P* = 0.80; *I* ^2^ = 0%).

#### Treatment effects on depression scores in BD

**Figure 3.**
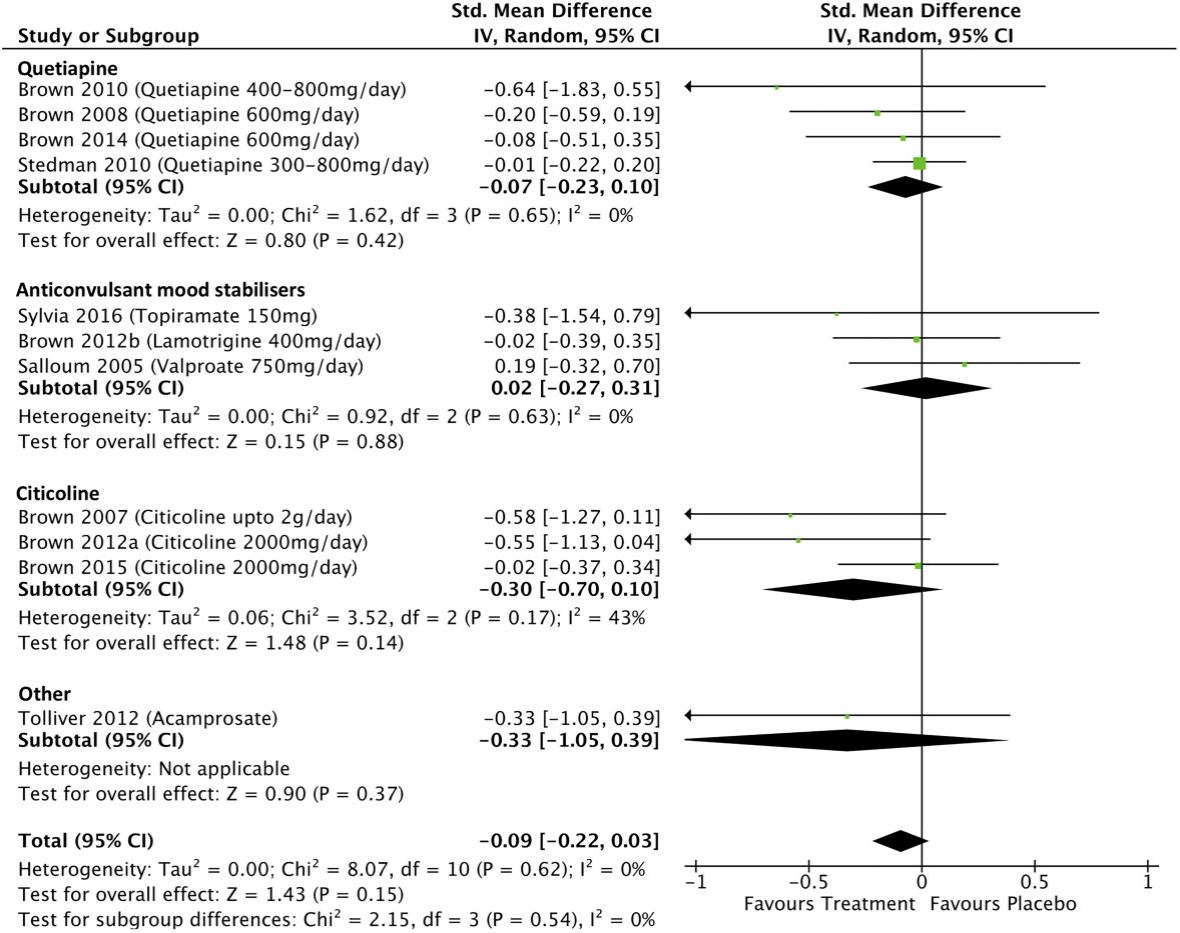
Effect of treatment on depression scores in bipolar disorder with addiction comobidity.

Depressive symptom score data from 11 studies that investigated the effects of treatment on depression scores in people with BD with addiction comorbidities were included in the meta-analysis. The overall pooled effect size (SMD) for these studies was −0.09 (95% CI, −0.22 to 0.03; *P* = 0.15; *I* ^2^ = 0%; see Figure 3). In the 6 studies where participants were reported as being in a predominantly depressed or mixed bipolar mood state,^25–27,29,36,56^ the pooled treatment effect size on depression scores was −0.03 (0.16 to 0.11; *P* = 0.67; *I* ^2^ = 0%). Only one study reported statistically significant improvements in end-of-trial depression scores: Brown et al. using citicoline 2,000 mg/day in people with BD or MDD and methamphetamine dependence (SMD Hamilton Depression Rating scale = −0.55).^35^

Four studies examined the effects of quetiapine on bipolar depression scores with a nonsignificant pooled effect size of −0.07 (95% CI, −0.23 to 0.1; *P* = 0.42; *I* ^2^ = 0%). Omitting one quetiapine study, which was assessed as weak quality,^34^ from the analysis changed the pooled effect size to −0.06 (95% CI, −0.22 to 0.11; *P* = 0.5; *I* ^2^ = 0%). Three studies examined the effects of anticonvulsant mood stabilizers on depression scores with a nonsignificant pooled effect size of 0.02 (95% CI, −0.27 to 0.31; *P* = 0.88; *I* ^2^ = 0%). Three studies examined the effects of citicoline on bipolar depression scores with a nonsignificant pooled effect size of −0.3 (95% CI, −0.70 to 0.1; *P* = 0.14; *I* ^2^ = 0%).

#### Treatment effects on MDD depression scores

**Figure 4.**
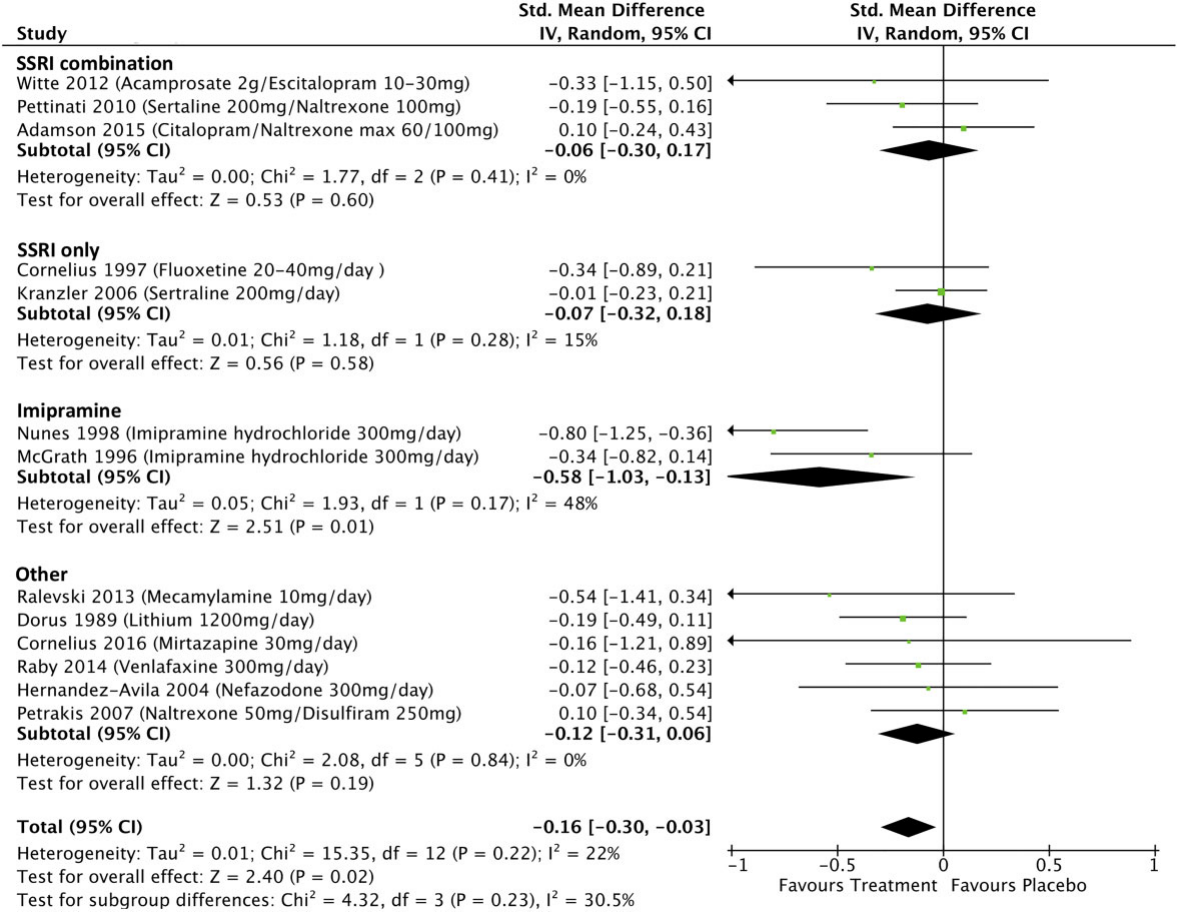
Treatment effects on depression scores in participants with major depressive disorder and comorbid addictions.

Data from 13 studies that investigated treatment effects on depression scores in participants with MDD with addiction comorbidities were included in the meta-analysis (see Figure 4). The overall pooled effect size was −0.16 (95% CI, −0.30 to −0.03; *P* = 0.02; *I* ^2^ = 22%; *P* = 0.22). Omitting 4 studies, which were assessed as weak quality,^39,42,45,47^ from the analysis changed the pooled effect size to −0.18 (95% CI, −0.36 to −0.0; *P* = 0.04; *I* ^2^ = 35%). Imipramine was associated with improvements in depression scores in 2 studies, in comorbid alcohol dependence and opiate dependence, respectively,^45,53^ with a significant pooled effect size of −0.58 (95% CI, −1.03 to −0.13; *P* = 0.01; *I* ^2^ = 48%). Selective serotonin reuptake Inhibitors (SSRI) treatments, either alone or in combination with relapse prevention medications such as naltrexone, had no significant effect on depressive symptoms in people with MDD and comorbid addictions (SSRI-only effect size −0.07; 95% CI, −0.32 to 0.18; *P* = 0.58; *I* ^2^ = 15%; SSRI combination effect size −0.06; 95% CI, −0.30 to 0.17; *P* = 0.60; *I* ^2^ = 0%).

A comparison of SSRI to non-SSRI antidepressant treatments in people with MDD and comorbid addictions found no significant differences in treatment effects (pooled effect size non-SSRI treatments −0.33; pooled effect size SSRI treatments −0.06; *P* = 0.011; see Supplemental Figure 2).

### Meta-Analysis: Treatment Effects on Addiction Measures

#### Effects of treatment on alcohol consumption

**Figure 5.**
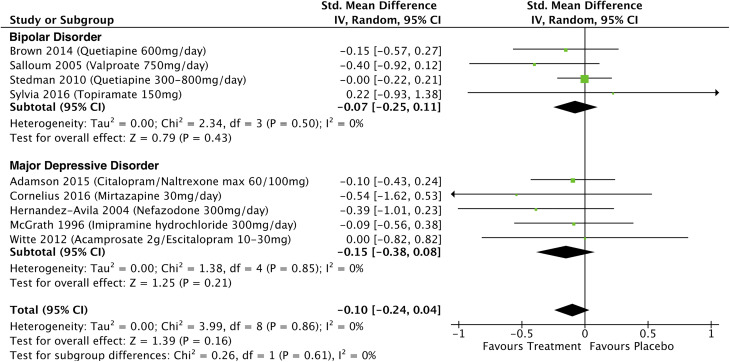
Effects of treatment on alcohol consumption in participants with mood disorders and comorbid addictions.

Alcohol consumption treatment effects in people with mood disorders were available for 9 studies (see Figure 5) and these had a nonsignificant overall pooled effect size of −0.07 (95% CI, −0.25 to 0.11; *P* = 0.43; *I* ^2^ = 0%) in BD and −0.15 (95% CI, −0.38 to 0.08; *P* = 0.21; *I* ^2^ = 0%) in MDD. In BD, Salloum et al. found a significant reduction in alcohol consumption associated with divalproex sodium treatment.^25^ In MDD, Pettinati et al. found a significant reduction in alcohol consumption and abstinence associated with sertraline and naltrexone treatment,^38^ Hernandez-Avila et al. reported a significant reduction in heavy drinking days associated with nefazodone treatment,^43^ Cornelius et al. found a reduction in total alcohol consumption over the course of the trial associated with fluoxetine treatment,^49^ and Petrakis et al. found a significant reduction in alcohol consumption and more consecutive days of abstinence associated with naltrexone or disulfiram treatment.^40^

#### Effects of treatment on alcohol abstinence

**Figure 6.**
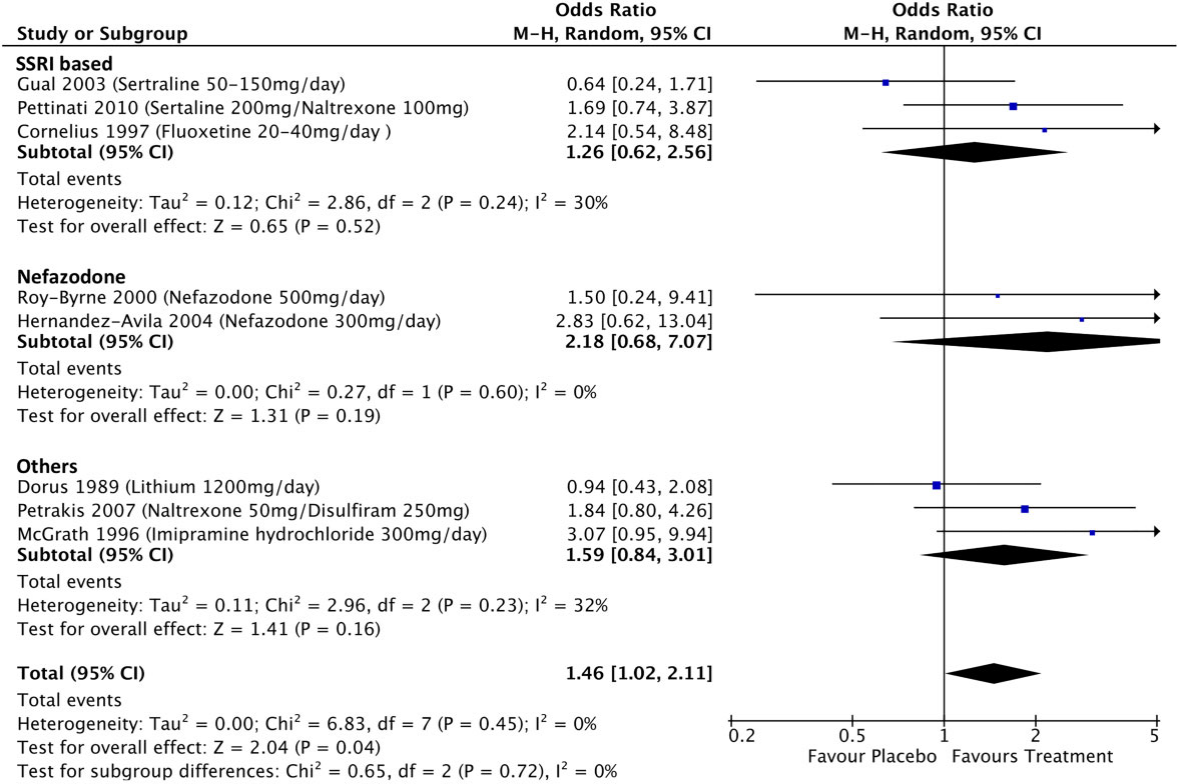
Effects of treatment on alcohol abstinence in participants with major depressive disorder and comorbid alcohol use disorder.

Treatment effects on alcohol abstinence was available for 8 studies in MDD but none for BD (see Figure 6); these studies showed a pooled *OR* for abstinence of 1.46 associated with treatment (95% CI, 1.02 to 2.11; *P* = 0.04; *I* ^2^ = 0%), with the highest *OR* of 3.1 associated with imipramine treatment.^45^

#### Effects of treatment on cocaine or opiate abstinence

**Figure 7.**
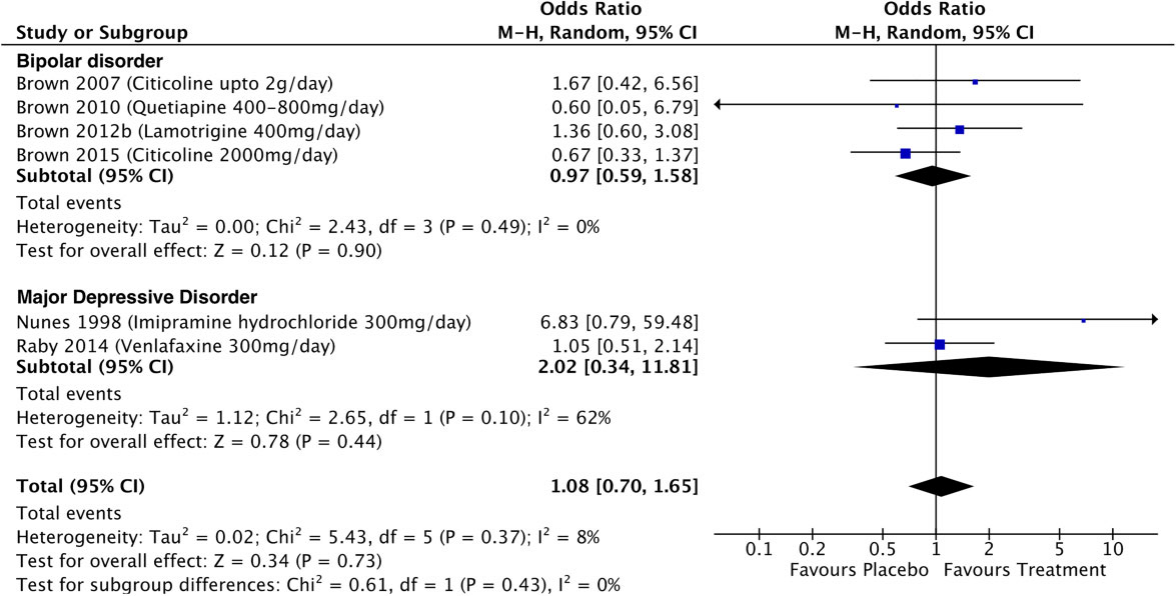
Effects of treatment on end-of-trial negative urine drug screens in participants with mood disorders and comorbid addictions.

Data on treatment-related effects on cocaine abstinence were available for 4 studies in BD, 1 study on cocaine abstinence in MDD, and 1 on opiate abstinence (see Figure 7). These studies used a negative end-of-study urine drug screen as a marker of abstinence. For the bipolar cocaine studies, the pooled *OR* of abstinence was 0.97 (95% CI, 0.59 to 1.58; *P* = 0.9; *I* ^2^ = 0%). Cocaine and opiate consumption were not reported consistently across studies, and so a meta-analysis of the effects of treatment on consumption was not possible.

#### Meta-analysis: Participant dropout and AEs

**Figure 8.**
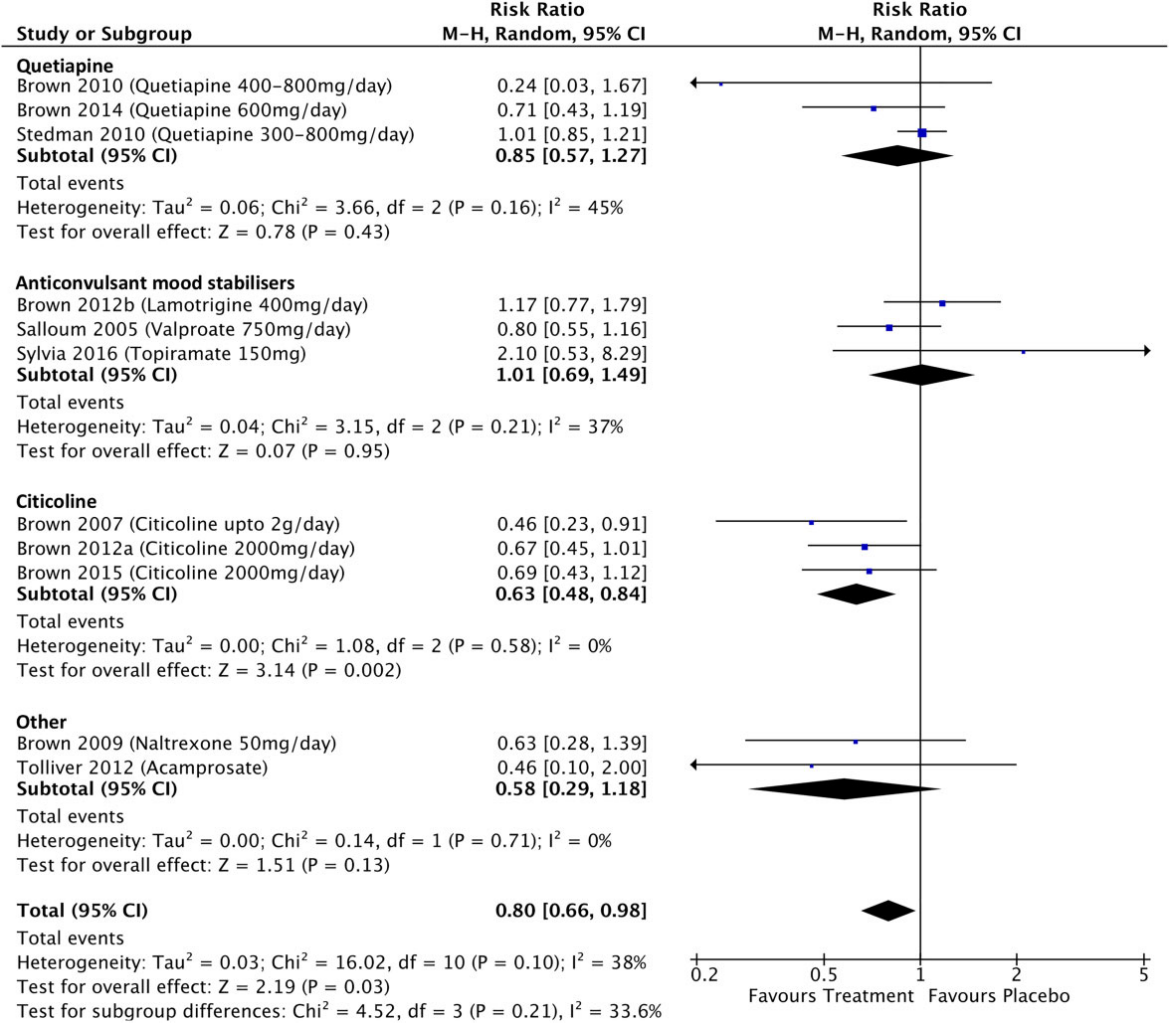
(A) Risk of treatment-associated dropout in participants with bipolar disorder. (B) Risk of treatment-associated dropout in participants with major depressive disorder.

Participant dropout data were available for 11 BD studies and 16 MDD studies (see Figure 8). For the BD studies, the RR of treatment-associated participant dropout was 0.80 (CI, 0.66 to 0.98; *P* = 0.03), significantly lower than those treated with placebo. There were no differences in the RR of dropout associated with quetiapine (RR, 0.85; CI, 0.57 to 1.27; *P* = 0.43), or anticonvulsant mood stabilizers (RR, 1.01; CI, 0.69 to 1.49; *P* = 0.95) compared with placebo, but citicoline was associated with a significantly lower risk of participant dropout (RR, 0.63; CI, 0.48 to 0.84; *P* = 0.002). For MDD studies, the RR of treatment-associated participant dropout was 1.10 (CI, 0.94 to 1.3; *P* = 0.24). There were no significant differences in the RRs of treatment-associated participant dropout associated with any specific drug type compared to placebo, with the exception of venlafaxine which was associated with a higher risk of dropout at a trend significance level (RR, 1.41; CI, 0.96 to 2.08; *P* = 0.08). The RR of treatment-associated dropout was significantly lower for the bipolar studies than the MDD studies (*P* = 0.01).

**Figure:**
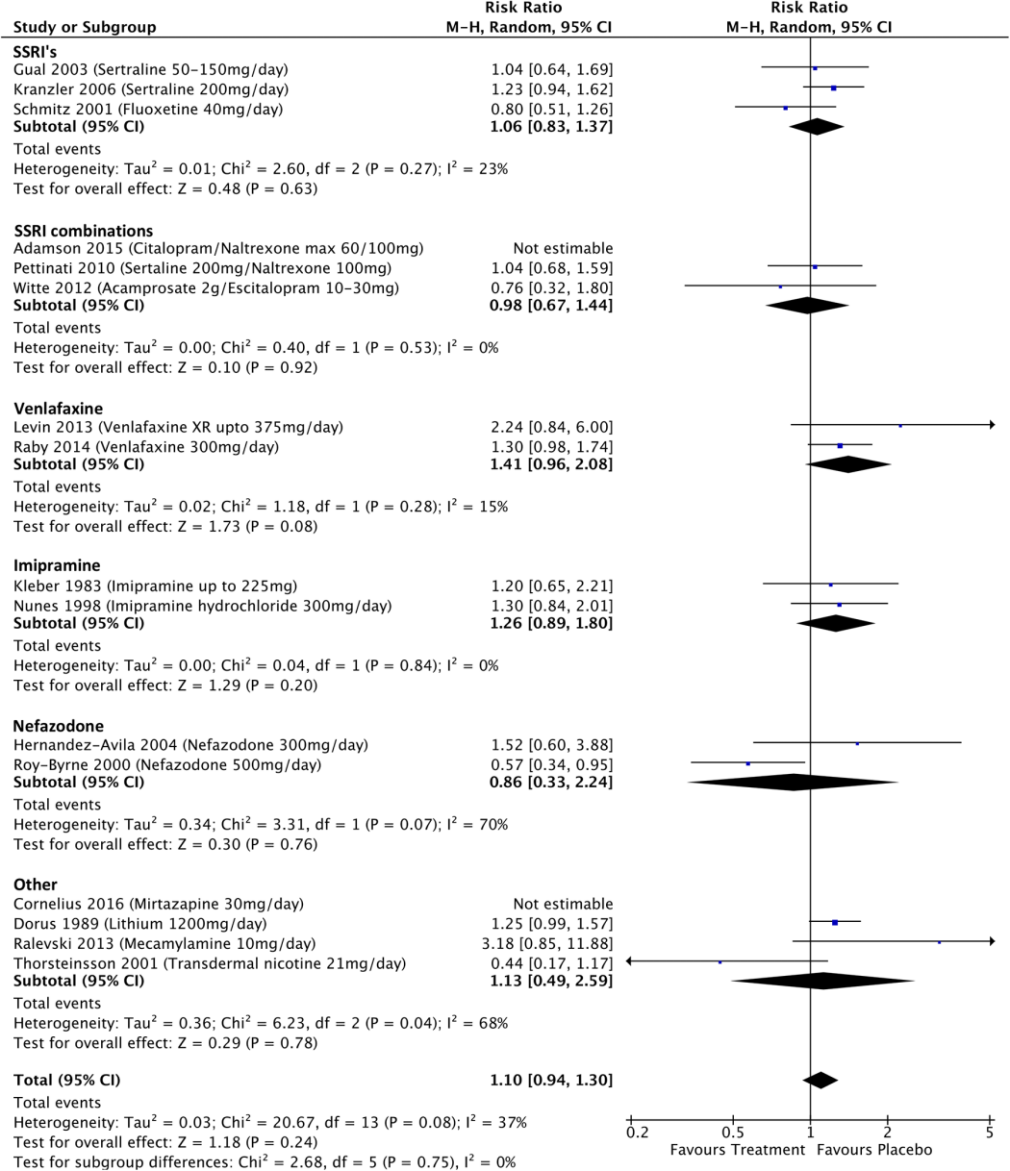


In all, 9 BD studies and 13 MDD studies reported rates of SAEs. There were no significant differences in treatment-related risks of SAEs (BD-RR, 0.92; CI, 0.52 to 1.64; *P* = 0.78; MDD-RR, 1.04; CI, 0.66 to 1.63; *P* = 0.87; see Supplemental Figure 3). A high rate of SAEs (RR > 10) was reported in a venlafaxine MDD study.^54^ In all, 10 BD studies and 9 MDD studies reported rates of psychiatric AEs with an RR of 1.06 (CI, 0.43 to 2.63; *P* = 0.90) and 1.89 (CI, 0.85 to 4.21; *P* = 0.12), respectively (see Supplemental Figure 4). High risk rates of psychiatric AEs (RR > 5) were found in the lamotrigine BD study^36^ and venlafaxine MDD study.^55^

## Discussion

In this study, we have identified differences in the efficacy of treatments for people with mood disorders with addiction comorbidity which have important implications for clinical practice. We found that pharmacological therapy was significantly more effective than placebo for improving manic symptoms in BD and depressive symptoms in MDD but importantly was not effective for improving depressive symptoms in BD. Pharmacological treatment significantly improved the odds of alcohol abstinence in MDD but had no effects on alcohol consumption or opiate abstinence in people with mood disorders. All of the significant effects on mood or addiction symptoms associated with pharmacological treatment identified by this meta-analysis were small in magnitude (SMD < −0.5) with the exception of imipramine for MDD and comorbid addictions where we found a medium effect size (SMD = −0.58). Although other reviews in the field have been published,^57–61^ this is, to our knowledge, the most comprehensive study investigating pharmacological treatments for people with mood disorders and addiction comorbidity.

Quetiapine was the medication most commonly studied for the treatment of people with BD with addictions comorbidity and was investigated in 4 studies. Three of these studies were of participants with comorbid alcohol addiction,^26,27,29^ 1 was of participants with comorbid cocaine dependence,^34^ and all 4 studies recruited participants who were not currently abstinent. The focus on quetiapine is not surprising given the robust evidence for its efficacy in treating both manic and depressive symptoms reported from studies of people with BD without addiction comorbidity. From our meta-analysis however, quetiapine treatment effects in people with BD and addiction comorbidity were markedly different from those reported in the nonaddicted bipolar population. Quetiapine did reduce mania scores in all 4 studies included in the meta-analysis with a significant but small pooled effect size of 0.23. However, critically quetiapine had no significant effect on depressive symptoms in BD with a pooled effect size of 0.07. These effect sizes associated with quetiapine treatment are lower than those reported in a nonaddicted BD population; 0.40 for the effect on manic symptoms^62^ and 0.29 for the effect on depressive symptoms.^63^ We would suggest that further studies investigate the biological and psychosocial factors that mediate differences in quetiapine efficacy between addicted and nonaddicted participants in bipolar depression. Our results support the use of quetiapine for the treatment of manic symptoms in people with BD and addiction comorbidity, although clinicians should be aware of smaller effect sizes, but they question the rationale for using quetiapine for the treatment of depression in people with BD and addiction comorbidity.

Of the other treatments used in BD, anticonvulsant mood stabilizers such as valproate and lamotrigine had no significant effects on mania or BD symptoms. Citicoline, an unregulated nootropic supplement, had promising albeit nonsignificant effects on depression scores but minimal effects on mania scores.^35,56^ Lithium was found by one study to have a significant effect on manic and depressive symptoms in people with BD and gambling disorder.^37^ It is particularly surprising that, although lithium is a key mood stabilizer for the treatment of BD, there have been no placebo controlled RCTs that we could find which have investigated lithium treatment effects in people with BD and alcohol or other substance use disorder comorbidities.

None of the BD studies with available outcome data for the meta-analysis showed significant treatment effects on alcohol consumption or cocaine/heroin abstinence. From the systematic review, citicoline was reported as significantly reducing cocaine use particularly at the beginning of the trial,^56^ lamotrigine was associated with a significant decrease in dollars spent on cocaine but had no effect on cocaine abstinence,^36^ valproate with a reduction in heavy alcohol drinking days,^25^ and lithium on pathological gambling symptoms.^37^ None of these results have been replicated in other studies. The medications used to treat BD with addiction comorbidities were generally well tolerated, with the risk of SAEs and psychiatric AEs being overall similar to that of placebo, and interestingly, we found a significantly overall lower risk of dropout associated with pharmacological treatment compared to placebo.

For MDD, the meta-analysis clearly showed that the treatment with the greatest effect on depressive symptoms was imipramine with a significant pooled effect size of 0.58. Although this presents a clear rationale for using imipramine as a treatment for people with MDD and addiction comorbidity, we would highlight that clinicians should consider the risks and benefits of its use for individual patients, particularly for those at risk of overdose. The meta-analysis found that no other type of pharmacological treatment significantly improved depressive symptoms in MDD. SSRI treatments either as monotherapy, or in combination with anti-addiction or relapse prevention medications, had no significant effect on depression scores with pooled effect sizes of 0.06 and 0.07, respectively. The lack of efficacy of SSRI’s found in our study, which confirms the findings of a previous meta-analysis,^58^ challenges the clinical rationale for using SSRIs for the treatment of depression in people with MDD and addiction comorbidity. Only one study investigated the effect of lithium in people with MDD and addiction comorbidity and this was in people recovering from alcohol dependence abstinent for at least 3 weeks. This study found no significant effects on mood or addiction measures, although this finding should be considered in the context that it is possible that depressive symptoms experienced by participants may have been secondary to alcohol dependence.^24^

Our meta-analysis found that no medications were significantly more effective than placebo in reducing alcohol consumption in people with MDD or improving the odds of abstinence from alcohol or other substances. However, several studies reported improvements in measures of alcohol addiction in the systematic review. A combination of sertraline and naltrexone improved alcohol abstinence rates in 1 trial,^38^ naltrexone or disulfiram treatment was associated with significantly fewer drinking days per week and more consecutive days of abstinence,^40^ nefazodone treatment was associated with a reduction in heavy alcohol drinking days,^43^ and fluoxetine treatment in reducing total trial alcohol consumption.^49^ Given the wide variety of addiction outcome measures used in these studies and the lack of replication of findings, it is difficult to interpret the impact that these findings have on treating addiction symptoms in people with mood disorders. Treatments for MDD with addiction comorbidity were overall well tolerated with no significant increase in rates of SAEs or psychiatric AEs associated with pharmacological treatments. Venlafaxine was associated with increased rates of both SAEs and psychiatric AEs in one study.^55^ Overall participant dropout rates for treatments for MDD with addiction comorbidity were not significantly different from placebo but interestingly were significantly higher than in BD.

There are a number of limitations of this review and for the field in general. Given the high prevalence and clinical challenge of addiction comorbidity in mood disorders, there were a surprisingly small number of high-quality, well-powered RCTs available. This may in part be due to people with mood disorders and addictions comorbidity being excluded from participation in clinical trials.^64^ The low number of RCTs was particularly surprising in BD given the markedly higher prevalence rates of addictions reported. It is also important to note that most studies in BD have been conducted by a small number of research groups which may limit independent verification of treatment effects. The lack of statistically significant results reported may partly be due to the small sample sizes used by most studies and potentially significant effects may have been missed due to low statistical power. However, the largest study included in this review, which had a sample size of 328, failed to detect any significant medication effects, suggesting that low power alone does not explain these findings.^26^ Nonetheless, larger trials would be very welcome. An important caveat for the interpretation of this study’s findings is that we found evidence of possible publication bias, where studies with negative findings may not be published. This was particularly evident for studies with small participant numbers or studies in BD. Nonpublication of negative findings may overexaggerate effect sizes associated with pharmacological treatment^65^ and our findings should be interpreted with this in mind. The identification of publication bias identified by our study underlines the need for all RCTs investigating pharmacological treatments for mood disorders and comorbid addictions to be published, supported by robust use of clinical trial registries.

A challenge for the field is that treatment effects reported by studies may have been masked by comparatively large placebo effects and by combining pharmacological treatments with psychological treatments. Placebo effects are a common finding in mood disorder clinical trials,^66^ and every trial included in this review reported an improvement in mood symptoms in the placebo arm, with many also reporting reductions in substance use associated with placebo treatment. Significant placebo effects have been previously identified in people with MDD/dysthymic disorder and comorbid alcohol use disorders but interestingly these placebo response rates were not significantly greater than those found in nonaddicted MDD/dysthymic disorder populations.^58^ Placebo effects potentially mask improvements that might otherwise have been attributable to medication. We would suggest that future studies consider using an enriched clinical trial design, such as the sequentially parallel design, which may reduce placebo responses.^67^ Importantly, some trials also used psychological therapies for both active and placebo arms. Cognitive behavioral therapy was most commonly used; other techniques used included motivational interviewing and relapse prevention therapy. There is good evidence that psychological therapies can be effective in treating both mood disorders and addictions,^68^ and so it is possible that medication effects in many of the included trials were masked by the effectiveness of psychological therapies.

A real challenge in conducting this meta-analysis and comparing treatment effects was the wide range of measurement scales used across studies, particularly for assessing substance use. Many studies focused on abstinence as the primary substance use outcome measure, which may fail to capture important improvements in substance use that fall short of full abstinence criteria. A wide variety of consumption outcomes were used across studies such as consumption per day or per week or number of days/week or weeks/month in which substances were used. This made the assessment of overall treatment effects on continuous substance outcomes difficult. Another challenge was variation in the mood state of participants treated in the trials identified. This could mean that effects associated with drug treatments in one mood state such as depression are lost or diluted as these results are merged with those from other mood states such as mania. With a few exceptions, most studies also excluded more severely depressed patients due to safety concerns. Suicidal ideation, for example, was frequently cited as an exclusion criterion. By contrast, one study that found promising results included participants experiencing a severe major depressive episode with high rates of suicidal ideation.^49^ It would be interesting to explore whether medication effects become more significant for more severely depressed patients. This would also be of great relevance to the clinical management of patients with these comorbidities, many of whom experience severe symptoms. Finally, a number of studies, particularly in MDD, did not clearly report end-of-study mood and addiction scores either in the research paper or in databases such as clinicaltrials.gov. We attempted to contact research groups where data were missing with little success which meant that 7 studies were not included in the meta-analysis.

Our study highlights the real clinical need for further robust, randomized placebo-controlled trials of treatments for mood disorder and addiction comorbidity with larger sample sizes and greater statistical power. We would suggest that inclusion criteria should be extended to include severely depressed patients, including where ethically permissible those with suicidal ideation, as it is in these cases that urgent clinical management is needed. Where ethical considerations allow, minimizing concomitant psychological therapies in trials might also help to unmask the impact of therapies under investigation. There is also a real need to conduct studies of participants in one mood state and to replicate treatment effects so that we can be sure that treatment effects generalize and are not false-positive findings. The challenges we found in comparing treatment effects also underline the importance of studies using the same mood and addiction outcome scores which are clearly reported. We would suggest that agreeing a consensus approach to mood and addiction outcome measures, including standard abstinence and substance consumption measures, for clinical trials of people with mood disorders and comorbid addictions is a key step in advancing the field. Finally, there is an exciting opportunity to use complementary or potentially alternative approaches to clinical trial methodologies, such as machine-learning analyses of electronic health records, to estimate pharmacological efficacy, particularly for those high-risk participants who may be excluded from current clinical trials.^69^

In conclusion, despite the challenges posed by small sample sizes and heterogeneity of results, this study provides valuable insights into the best ways of treating people with mood disorders and comorbid addictions. For BD, quetiapine is effective for the treatment of mania but importantly not for bipolar depressive symptoms. For MDD, our study confirmed the comparative effectiveness of imipramine for the treatment of depressive symptoms over other treatment types found by a previous meta-analysis^58^ and the lack of efficacy of SSRI-based treatment approaches. There was no convincing evidence that any specific treatment substantially improved addiction measures. Our study reinforces the urgent need for further well-powered high-quality clinical trials of treatments for mood disorder and comorbid addictions and for research that provides a greater understanding of neurobiological mechanisms so that treatments can be rationally designed and targeted.

## Supplemental Material

Supplementary_Material - Pharmacological Treatment of Mood Disorders and Comorbid Addictions: A Systematic Review and Meta-Analysis: Traitement Pharmacologique des Troubles de L’humeur et des Dépendances Comorbides: Une Revue Systématique et une Méta-Analyse Click here for additional data file. Supplementary_Material for Pharmacological Treatment of Mood Disorders and Comorbid Addictions: A Systematic Review and Meta-Analysis: Traitement Pharmacologique des Troubles de L’humeur et des Dépendances Comorbides: Une Revue Systématique et une Méta-Analyse by Paul R. A. Stokes, Tahir Jokinen, Sami Amawi, Mutahira Qureshi, Muhammad Ishrat Husain, Lakshmi N. Yatham, John Strang and Allan H. Young in The Canadian Journal of Psychiatry
