# Supplementary material for: Pharmacological Treatment of Mood Disorders and Comorbid Addictions: A Systematic Review and Meta-Analysis: Traitement Pharmacologique des Troubles de L’humeur et des Dépendances Comorbides: Une Revue Systématique et une Méta-Analyse
Source: Can J Psychiatry. 2020 Apr 17;65(11):749–69. doi: 10.1177/0706743720915420 (PMC7564307; doi:10.1177/0706743720915420)
Supplement: Supplementary_Material - Pharmacological Treatment of Mood Disorders and Comorbid Addictions: A Systematic Review and Meta-Analysis: Traitement Pharmacologique des Troubles de L’humeur et des Dépendances Comorbides: Une Revue Systématique et une Méta-Analyse [file Supplementary_Material.pdf]

First Author	Year of publication	Consensus global Quality Assessment score
Adamson	2015	1
Brown	2009	1
Brown	2010	3
Brown	2014	2
Brown	2015	2
Brown	2007	2
Brown	2008	2
Brown (citicoline)	2012a	2
Brown (lamotrigine)	2012b	2
Cornelius	1997	2
Cornelius	2016	3
Dorus	1989	2
Gual	2003	2
Hernandez-Avila	2004	1
Hollander	2005	2
Kleber	1983	2
Kranzler	2006	3
Levin	2013	1
McGrath	1996	3
Nunes	1998	1
Petrakis	2007	1
Pettinati	2010	1
Raby	2014	1
Ralevski	2013	3
Roy-Burne	2000	3
Salloum	2005	1
Schmitz	2001	2
Stedman	2010	2
Sylvia	2016	2
Thorsteinsson	2001	3
Tolliver	2012	1
Witte	2012	2

Supplementary Table 1: Consensus quality assessment scores for included mood disorder with addiction co-morbidity treatment studies

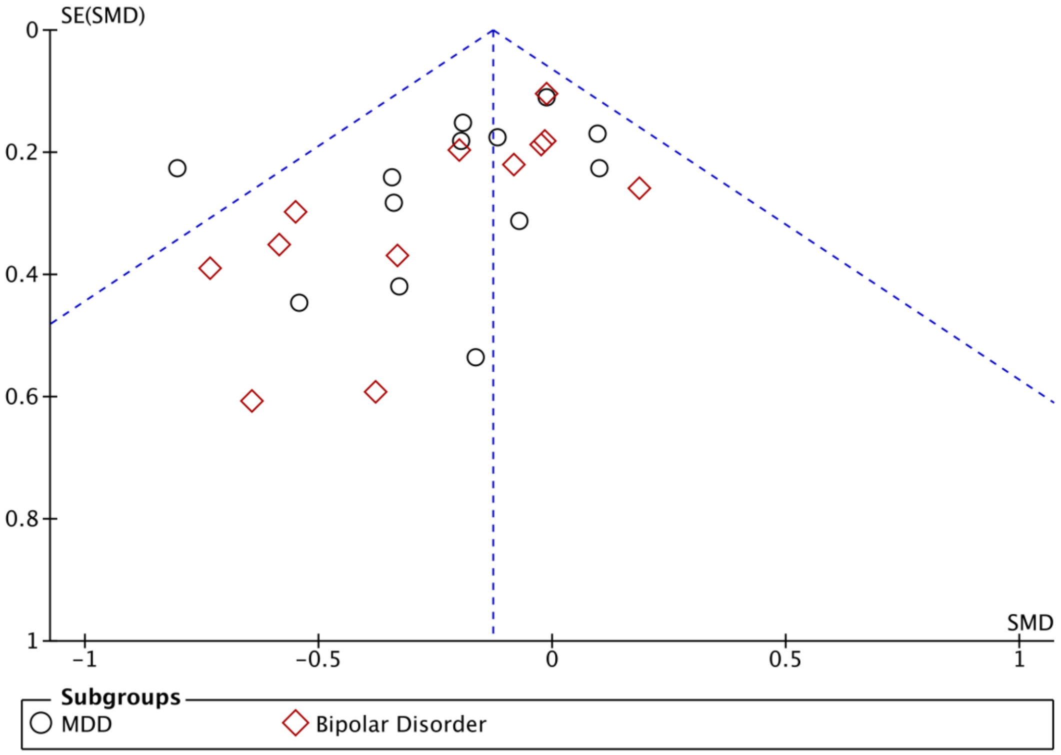

Supplementary Figure 1: Funnel plot of placebo controlled randomised clinical trials investigating effects of pharmacological therapies on depression scores in people with mood disorders and co-morbid addictions.

Study	Std. Mean Difference IV, Random, 95% CI
**Non-SSRI**	
Nunes 1998 (Imipramine hydrochloride 300mg/day)	−0.80 [−1.25, −0.36]
McGrath 1996 (Imipramine hydrochloride 300mg/day)	−0.34 [−0.82, 0.14]
Cornelius 2016 (Mirtazapine 30mg/day)	−0.16 [−1.21, 0.89]
Raby 2014 (Venlafaxine 300mg/day)	−0.12 [−0.46, 0.23]
Hernandez−Avila 2004 (Nefazodone 300mg/day)	−0.07 [−0.68, 0.54]
**Subtotal (95% CI)**	**−0.33 [−0.62, −0.03]**
Heterogeneity: $Tau^2 = 0.04$; $Chi^2 = 6.55$, df = 4 (P = 0.16); $I^2 = 39\%$	
Test for overall effect: Z = 2.18 (P = 0.03)	
**SSRI**	
Cornelius 1997 (Fluoxetine 20−40mg/day)	−0.34 [−0.89, 0.21]
Witte 2012 (Acamprosate 2g/Escitalopram 10−30mg)	−0.33 [−1.15, 0.50]
Pettinati 2010 (Sertaline 200mg/Naltrexone 100mg)	−0.19 [−0.55, 0.16]
Kranzler 2006 (Sertraline 200mg/day)	−0.01 [−0.23, 0.21]
Adamson 2015 (Citalopram/Naltrexone max 60/100mg)	0.10 [−0.24, 0.43]
**Subtotal (95% CI)**	**−0.06 [−0.21, 0.10]**
Heterogeneity: $Tau^2 = 0.00$; $Chi^2 = 2.95$, df = 4 (P = 0.57); $I^2 = 0\%$	
Test for overall effect: Z = 0.74 (P = 0.46)	
**Total (95% CI)**	**−0.18 [−0.35, −0.02]**
Heterogeneity: $Tau^2 = 0.02$; $Chi^2 = 13.30$, df = 9 (P = 0.15); $I^2 = 32\%$	
Test for overall effect: Z = 2.17 (P = 0.03)	
Test for subgroup differences: $Chi^2 = 2.54$, df = 1 (P = 0.11), $I^2 = 60.7\%$	

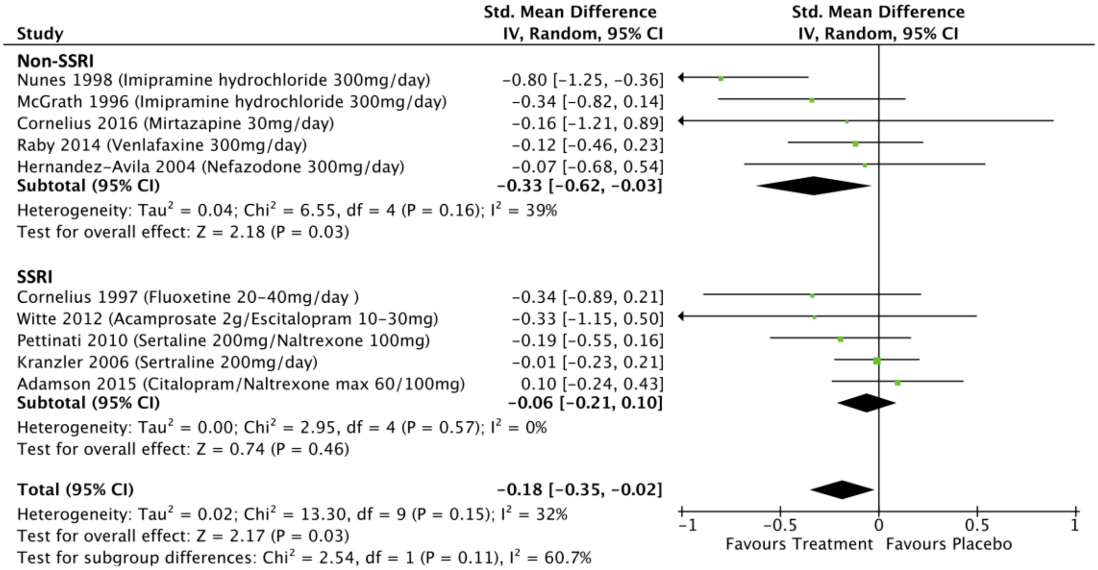

Supplementary Figure 2: Comparison of SSRI vs non-SSRI treatment effects on depression scores in participants with MDD and co-morbid addictions

Study or Subgroup	Risk Ratio M-H, Random, 95% CI
**Bipolar Disorder**	
Brown 2007 (Citicoline upto 2g/day)	0.46 [0.04, 4.68]
Brown 2008 (Quetiapine 600mg/day)	1.67 [0.42, 6.56]
Brown 2010 (Quetiapine 400-800mg/day)	Not estimable
Brown 2012a (Citicoline 2000mg/day)	0.18 [0.02, 1.48]
Brown 2012b (Lamotrigine 400mg/day)	1.30 [0.37, 4.57]
Brown 2014 (Quetiapine 600mg/day)	1.67 [0.42, 6.55]
Brown 2015 (Citicoline 2000mg/day)	0.63 [0.22, 1.80]
Salloum 2005 (Valproate 750mg/day)	Not estimable
Tolliver 2012 (Acamprosate)	Not estimable
**Subtotal (95% CI)**	**0.92 [0.52, 1.64]**
Total events	
Heterogeneity: $Tau^2 = 0.00$; $Chi^2 = 4.92$, df = 5 (P = 0.43); $I^2 = 0\%$	
Test for overall effect: Z = 0.27 (P = 0.78)	
**Major Depressive Disorder**	
Cornelius 1997 (Fluoxetine 20-40mg/day)	Not estimable
Cornelius 2016 (Mirtazapine 30mg/day)	Not estimable
Gual 2003 (Sertraline 50-150mg/day)	Not estimable
Kranzler 2006 (Sertraline 200mg/day)	1.34 [0.54, 3.30]
Levin 2013 (Venlafaxine XR upto 375mg/day)	Not estimable
Petrakis 2007 (Naltrexone 50mg/Disulfiram 250mg)	0.75 [0.26, 2.13]
Pettinati 2010 (Sertaline 200mg/Naltrexone 100mg)	0.89 [0.50, 1.60]
Raby 2014 (Venlafaxine 300mg/day)	13.40 [0.77, 233.08]
Ralevski 2013 (Mecamylamine 10mg/day)	Not estimable
Roy-Byrne 2000 (Nefazodone 500mg/day)	Not estimable
Schmitz 2001 (Fluoxetine 40mg/day)	Not estimable
Thorsteinsson 2001 (Transdermal nicotine 21mg/day)	Not estimable
Witte 2012 (Acamprosate 2g/Escitalopram 10-30mg)	1.14 [0.27, 4.78]
**Subtotal (95% CI)**	**1.04 [0.66, 1.63]**
Total events	
Heterogeneity: $Tau^2 = 0.02$; $Chi^2 = 4.30$, df = 4 (P = 0.37); $I^2 = 7\%$	
Test for overall effect: Z = 0.16 (P = 0.87)	
**Total (95% CI)**	**0.99 [0.70, 1.39]**
Total events	
Heterogeneity: $Tau^2 = 0.00$; $Chi^2 = 9.09$, df = 10 (P = 0.52); $I^2 = 0\%$	
Test for overall effect: Z = 0.07 (P = 0.95)	
Test for subgroup differences: $Chi^2 = 0.10$, df = 1 (P = 0.75), $I^2 = 0\%$	

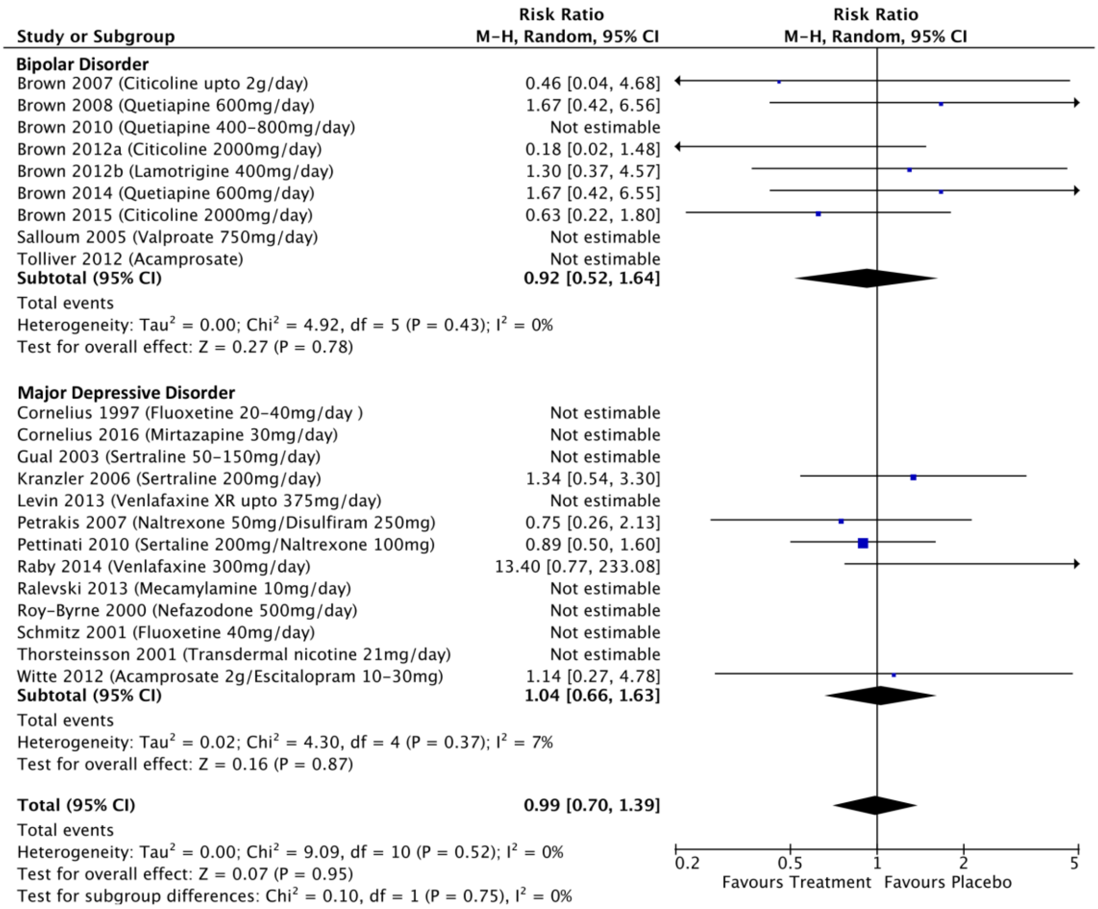

Supplementary Figure 3: Risk of Serious Adverse Events

Study or Subgroup	Risk Ratio M–H, Random, 95% CI
**Bipolar Disorder**	
Brown 2007 (Citicoline upto 2g/day)	0.44 [0.04, 4.57]
Brown 2008 (Quetiapine 600mg/day)	Not estimable
Brown 2010 (Quetiapine 400–800mg/day)	Not estimable
Brown 2012a (Citicoline 2000mg/day)	0.71 [0.05, 10.75]
Brown 2012b (Lamotrigine 400mg/day)	5.18 [0.63, 42.95]
Brown 2014 (Quetiapine 600mg/day)	2.00 [0.19, 21.26]
Brown 2015 (Citicoline 2000mg/day)	0.20 [0.01, 4.08]
Salloum 2005 (Valproate 750mg/day)	Not estimable
Stedman 2010 (Quetiapine 300–800mg/day)	0.35 [0.04, 3.37]
Tolliver 2012 (Acamprosate)	2.29 [0.23, 22.59]
**Subtotal (95% CI)**	**1.06 [0.43, 2.63]**
Total events	
Heterogeneity: Tau^2 = 0.00; Chi^2 = 5.58, df = 6 (P = 0.47); I^2 = 0%	
Test for overall effect: Z = 0.13 (P = 0.90)	
**Major Depressive Disorder**	
Adamson 2015 (Citalopram/Naltrexone max 60/100mg)	Not estimable
Cornelius 1997 (Fluoxetine 20–40mg/day)	Not estimable
Cornelius 2016 (Mirtazapine 30mg/day)	Not estimable
Gual 2003 (Sertraline 50–150mg/day)	Not estimable
Kranzler 2006 (Sertraline 200mg/day)	1.60 [0.58, 4.40]
Levin 2013 (Venlafaxine XR upto 375mg/day)	6.12 [0.76, 49.04]
Ralevski 2013 (Mecamylamine 10mg/day)	1.82 [0.19, 17.12]
Thorsteinsson 2001 (Transdermal nicotine 21mg/day)	Not estimable
Witte 2012 (Acamprosate 2g/Escitalopram 10–30mg)	0.92 [0.06, 12.95]
**Subtotal (95% CI)**	**1.89 [0.85, 4.21]**
Total events	
Heterogeneity: Tau^2 = 0.00; Chi^2 = 1.65, df = 3 (P = 0.65); I^2 = 0%	
Test for overall effect: Z = 1.55 (P = 0.12)	
**Total (95% CI)**	**1.47 [0.80, 2.67]**
Total events	
Heterogeneity: Tau^2 = 0.00; Chi^2 = 8.07, df = 10 (P = 0.62); I^2 = 0%	
Test for overall effect: Z = 1.25 (P = 0.21)	
Test for subgroup differences: Chi^2 = 0.87, df = 1 (P = 0.35), I^2 = 0%	

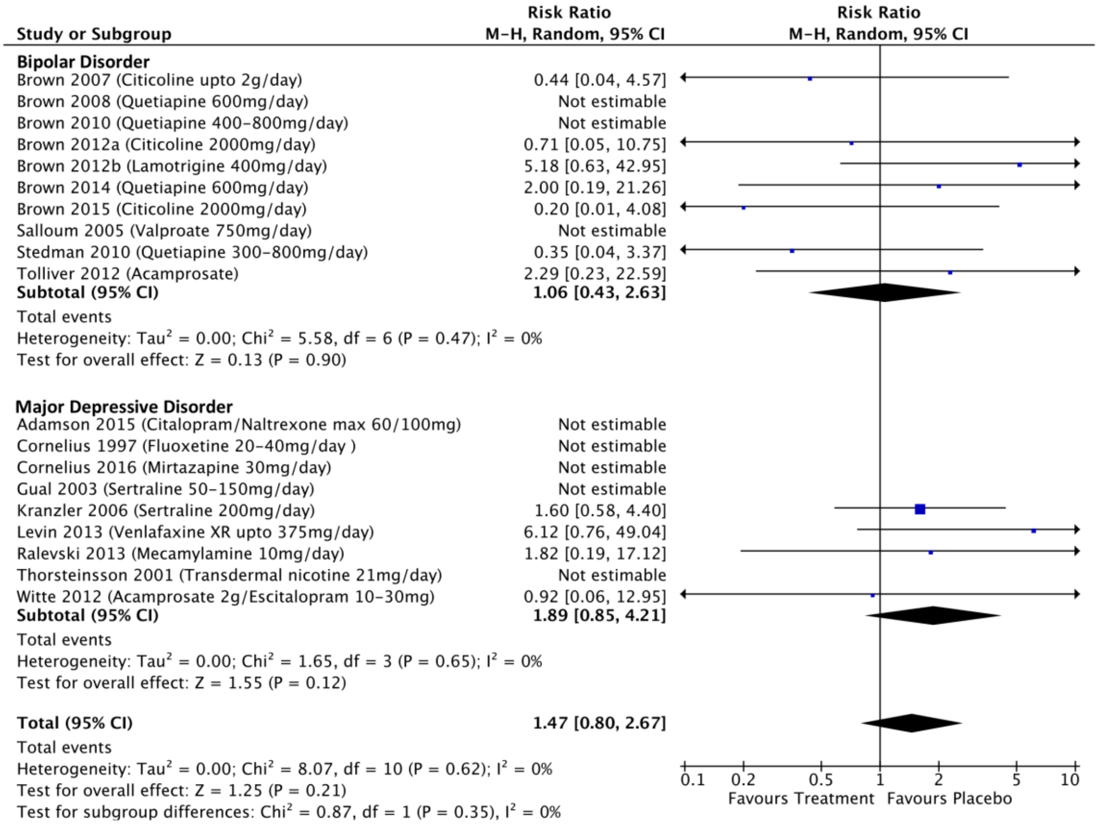

Supplementary Figure 4: Risk of psychiatric adverse events
